# Functional outcome and complications following reconstruction for Harrington class II and III periacetabular metastasis

**DOI:** 10.1186/1477-7819-13-4

**Published:** 2015-01-12

**Authors:** Piya Kiatisevi, Bhasanan Sukunthanak, Charoenchai Pakpianpairoj, Prasert Liupolvanish

**Affiliations:** Orthopaedic Oncology Unit, Institute of Orthopaedics, Lerdsin General Hospital, 190 Silom Rd., Bangrak, Bangkok, Thailand; Adult Reconstruction Unit, Institute of Orthopaedics, Lerdsin General Hospital, Bangkok, Thailand

## Abstract

**Background:**

Metastatic bone disease involving the acetabulum is a debilitating condition causing significant pain and disability for patients. Many methods of reconstruction have been described for treating Harrington class II and III lesions with different results and complications. Our objectives were to report functional results, implant survival and complications following reconstruction for Harrington class II and III periacetabular metastases by using anti-protusio cages, screws and joint replacement.

**Methods:**

We reviewed 22 patients undergoing acetabular reconstruction for metastatic disease. There were 5 Harrington class II and 17 class III lesions. Intralesional curettage, multiple screws and cemented total hip replacement were performed in all patients. Anti-protusio cages were used in 19 hips. No Steinmann pins were used. Sixteen patients died at a median survival time of 12 months (range, 4 to 28 months) after surgery. Six patients were alive at last follow-up at a median of 8 months (range, 3 to 15 months).

**Results:**

Postoperatively, the average ECOG score was improved from 3.1 to 1.7 and Visual Analog Scale was improved from 8.4 to 2.2. One patient developed hip dislocation and one patient developed superficial infection. The mean Musculoskeletal Tumor Society (MSTS) functional score was 70 (range, 27 to 87). There was no prosthetic loosening or revision. Twenty patients were able to walk. Eight patients became community ambulators, twelve became household ambulators and two were bed-bound.

**Conclusions:**

Good functional outcome and better ambulation could be expected following class II and III periacetabular reconstruction using anti-protusio cages, screws and cemented hip replacement. Few complications were noted and manageable. Although most of these patients with metastatic disease had limited life expectancies, their quality of life would be improved with appropriate patient selection and surgical reconstruction.

## Background

Metastatic bone disease involving the acetabulum is a debilitating condition causing significant pain and disability for patients. Large lesions causing impending or pathological fracture result in considerable patient dependency and disability. In some patients with reasonable life expectancy, surgical reconstructions are useful and efficient to relieve pain, restore function and create independent ambulation. When using the Harrington classification [1], class I metastatic lesions are usually reconstructed successfully with conventional cemented total hip replacement. However, in class II and class III lesions, various methods of Harrington modification reconstruction have been described in the medical literatures with different results and complications. We present our experience of class II and III periacetabular reconstruction using anti-protusio cage, multiple screws and cement total hip replacement in terms of functional result and complications.

## Methods

We retrospectively reviewed 22 hips in 22 consecutive patients who had Harrington class II and III lesion reconstruction for metastatic bone disease involving the acetabulum between January 2003 and March 2013. There were 14 men and 8 women with a mean age of 54 years (range, 33 to 71 years). The original primary tumors consisted of lung carcinoma in eight patients, thyroid carcinoma in three, hepatocellular carcinoma in two, endometrial carcinoma in two, cervical carcinoma in one, breast carcinoma in one, cholangiocarcinoma in one, nasopharyngeal carcinoma in one, thymic carcinoma in one, renal cell carcinoma in one and unknown primary origin in one patient. Five of the patients had class II lesions where the medial wall of the acetabulum is deficient and 17 patients had class III lesions where the lateral wall, superior acetabular dome and/or medial wall is deficient. Twelve patients presented with pathological fracture of the acetabulum and six patients had a central protusion of the femoral head. Four patients had either chemotherapy or radiation therapy for their primary malignancy prior to reconstruction. Eighteen patients had not had any treatment at the time of initial evaluation.

Patients were preoperatively evaluated with a complete history and physical examination. Plain radiographs of the pelvis and femur, technetium^99m^ bone scintigraphy, computed tomography (CT) scans of the chest, abdomen and pelvis and magnetic resonance imaging (MRI) were routinely performed in every case. CT-guided core needle biopsies were performed in patients with single lesion from technetium^99m^ bone scintigraphy and in whom the diagnosis was not established. All patients underwent preoperative embolization, intended to reduce intraoperative blood loss, at 1 or 2 days prior to the operation.

**Figure 1:**
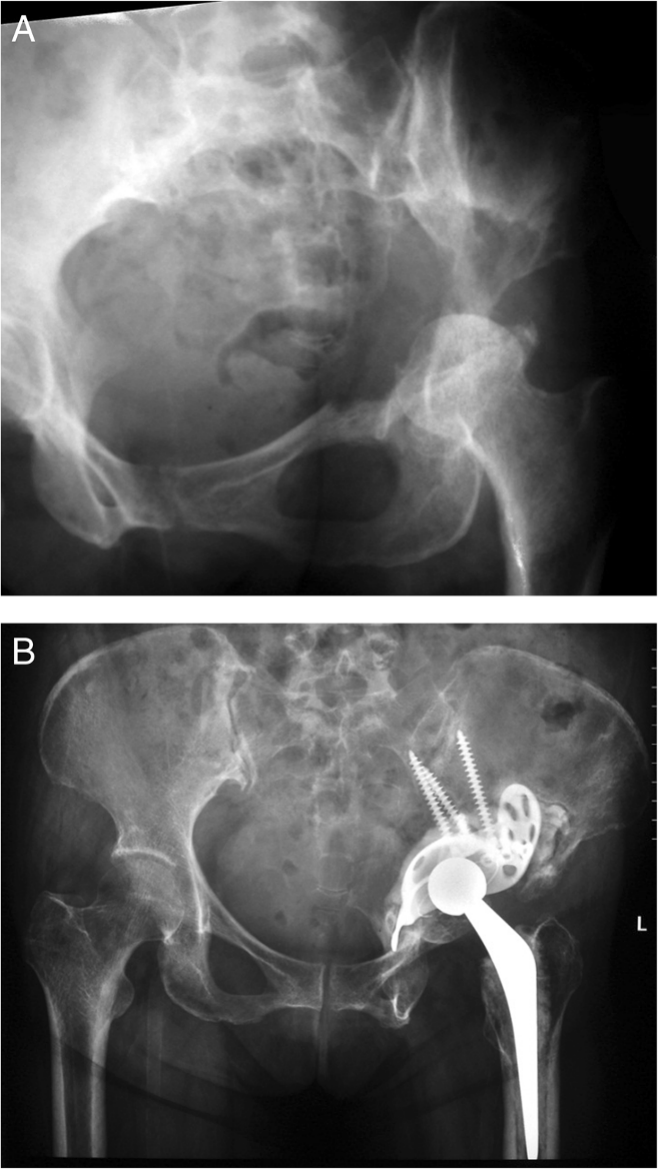
**Radiograph of left hip of a 53 year-old woman with class III periacetabular metastatic disease from thyroid carcinoma. (A)** preoperative radiograph showed a large lesion involving the superior dome and medial wall. **(B)** after reconstruction with anti-protusio cage, screws and total hip arthroplasty. The patient was able to walk without walking aid but died of her disease at 28 months after the surgery without any complication.

**Figure 2:**
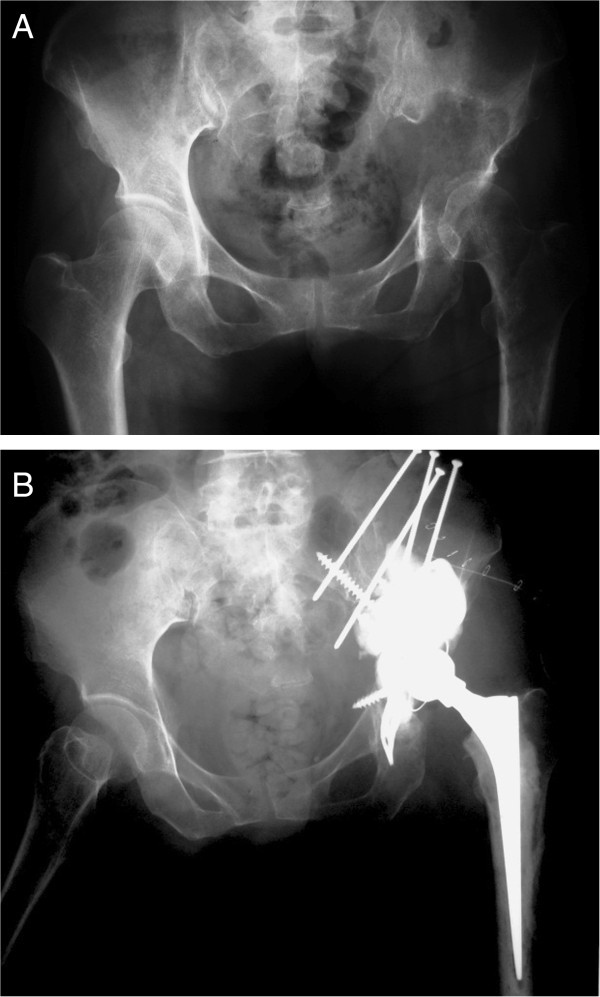
**Radiograph of left hip of a 58 year-old man with class III periacetabular metastatic disease from hepatocellular carcinoma. (A)** preoperative radiograph showed a large lesion involving the superior dome and medial wall. **(B)** after reconstruction with anti-protusio cage, screws and total hip arthroplasty. The patient was able to walk with a cane but died of disease at 12 months after the surgery without any complication.

**Figure 3:**
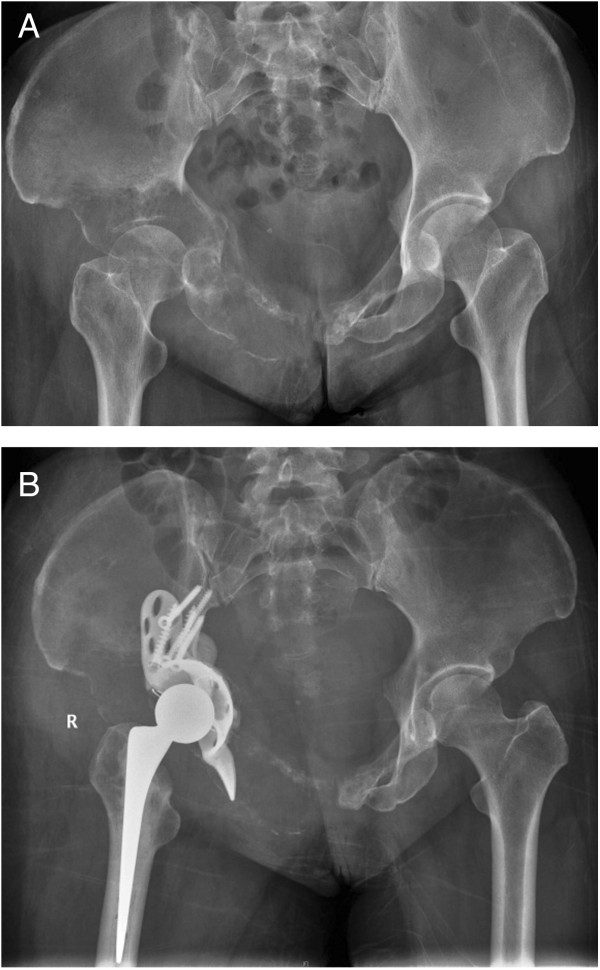
**Radiograph of left hip of a 58 year-old woman with class III periacetabular metastatic disease from lung carcinoma. (A)** preoperative radiograph showed a large lesion involving the superior dome and medial wall. **(B)** after reconstruction with anti-protusio cage, screws and total hip arthroplasty. The patient was able to walk with a cane and was still alive at 6 months after the surgery.

All operations were performed by a same group of surgeons. Depending on the main location of the tumor, the anterior iliofemoral approached was performed in 4 patients and the postero-lateral approach was performed in 18 patients. The operative procedure was intralesional removal of the metastatic lesion and cemented total hip arthroplasty. The Bursh-Schneider cages (Zimmer, Warsaw, IN, USA) were placed in 19 patients: 3 patients in class II and 16 patients in class III, and fixed with multiple screws aiming to catch good quality bone in the sacroiliac joint area. Fluoroscopy was utilized to confirm the position of the screws and ensure that the distal flanges were seated in the ischium. Steinmann pins were not utilized. However, long multiple screws were used instead of Steinmann pins in four cases in which the construct was not fully stable by using only cage and screws. The joints in the remaining three patients in whom the cage was not utilized were reconstructed with cementation, multiple screws and hip arthroplasty. The acetabular defect was filled entirely with Simplex® with erythromycin cement (Howmedica International, Limerick, Ireland) in all cases. After the cement was hardened, a ZCA cup (Zimmer, Warsaw, IN, USA) was cemented in the cage at 40° to 55° horizontal inclination and appropriate anterversion. A CPT stem (Zimmer, Warsaw, IN, USA) was cemented into the femur in standard fashion. A 32-mm Versys femoral head (Zimmer, Warsaw, IN, USA) was impacted to the stem prior to careful reduction of the hip (Figures 1, 2 and 3). The hip capsule was tightly repaired with number 2 Ethibond sutures. (Ethicon Inc., Somerville, NJ, USA) In two reconstruction cases where the hip capsule was removed with the tumor, a vascular graft was used to replace the original capsule and was tightly sutured with the remaining capsule until the graft and the remaining capsule circumferentially covered the hip joint.

Postoperative physical therapy was provided in all cases and the patients were allowed full weight-bearing as tolerated as early as possible. A medical oncologist was consulted to evaluate and treat with appropriate systemic adjuvant therapy. All patients received postoperative radiation to the operative site.

The 1993 version of the Musculoskeletal Tumor Society (MSTS) lower extremity score was used to determine functional outcome at the latest follow-up examination [2]. This score measures pain, function, emotional acceptance, supports, walking ability and gait. Each of these 6 variables was assessed on a 5-point scale, giving a maximum score of 30 points, which will be recorded as 100%. Visual Analog Scale (VAS), which ranges from 0 (no pain) to 10 (severe pain), was used to assess pain perception preoperatively and at the follow-up examination. We also used the Eastern Cooperative Oncology group (ECOG) performance status, which is a scale and criteria to assess how a patient’s disease is progressing, to assess how the disease affects the patient's daily living abilities [3]. The ECOG scores range from 0 (normal activity) to 4 (totally bed-ridden). Pre- and postoperative functional scores were compared using the Student's *t-*test or Wilcoxon rank-sum test after testing for normal distribution. Mean survival time and 95% confidence intervals were reported. All *P-*values were 2-sided (*P* < 0.05). Data were analyzed using SPSS statistical software version 11.5 (SPSS Inc, Chicago, IL, USA).

## Results

Most patients improved their ambulatory status after their operations. One household ambulator, sixteen bed-bound and five wheel-chair-bound patients became eight community and twelve household ambulators. Two patients remained bed-bound because of pain from metastatic disease at the lumbar spine. The average preoperative ECOG score of 3.1 (standard deviation (SD) 0.69) was improved when compared to the average postoperative ECOG score of 1.7 (standard deviation 0.82) (*P* < 0.001). Pain was reduced in every patient after surgery, with a mean score of 2.2 (SD 1.9) compared with a preoperative mean score of 8.4 (SD 0.7) (*P* < 0.001). Eight patients (36%) became independent and 12 (55%) needed partial assistance. The mean Musculoskeletal Tumor Society (MSTS) functional score in all patients was 70 (range, 27 to 87).

There was no statistical difference in mean estimated blood loss between patients with class II lesions, which was 2,740 ml (range 500 to 5,200 ml), and patients with class III lesions, which was 2,560 ml (range 900 to 8,000 ml), (*P* = 0.87). There was also no statistical difference in the median number of units of packed red cell transfusion required between patients with class II (5.2 units (range 2 to 11)) and with class III lesions (4.5 units (range 3 to 11)), (*P* = 0.75).

We had one (5%) patient who developed a hip dislocation after changing her body position at 1 week postoperatively. The posterior approach had been utilized in this patient. The patient was treated with closed reduction and was followed for 24 months with no sign of re-dislocation. There was one (5%) patient who developed superficial infection but this was treated successfully with wound irrigation and intravenous antibiotics. One patient (5%) experienced permanent foot-drop postoperatively. Two (9%) deep vein thrombosis patients were treated with heparin and followed by oral anticoagulants. No patient developed pulmonary embolism. There was no implant loosening, implant revision required or perioperative death.

During the review period, 6 patients (27%) were alive with evidence of disease at a mean follow-up of 8 months (range 3 to 15). Sixteen patients (72%) died of their disease at a mean follow-up of 12 months (range 4 to 28). The median survival time in the entire population was 11 months (range 3 to 28).

## Discussion

Surgical treatment for metastatic disease involving the acetabulum, especially in Harrington class II and III cases, is difficult and challenging due to its complex anatomy, proximity to major neurovascular structures and the possibility of massive intraoperative bleeding. Pathological fractures causing pelvic discontinuity and protusio acetabuli are not uncommon and complicate the surgical treatment as well as increase the risk of perioperative morbidity. With a relatively short life expectancy in this group of patients, a proper and stable reconstruction is needed with the goals of improving pain, function, ambulation and independence.

Periacetabular reconstruction for class II and class II lesions needs restoration of structural integrity of the medial wall, superior dome and lateral wall to allow placement of the acetabular components. Many surgical techniques have been proposed for acetabular reconstruction for class II and III lesions. Harrington [1] described good results with no loosening of implants by using a protusio ring with or without mesh in class II and adding threaded-Steinmann pins in class III lesions. Ho [4] reported good reconstruction results of class III lesions using the ‘rebar’ method which was fixation only using screws and cement. There was no implant failure due to loosening of the acetabular cup or cement. Clayer [5] also reported good reconstruction results of class II and III lesions by using protrusio cages without the use of supplemental fixation by Steinmann pins. There was only one cage that avulsed with loss of fixation due to disease progression. Hoell [6] reported good results of reconstruction of class II and III lesions by using the Burch-Schneider cage. Of 15 patients from their series, 2 patients developed significant radiologic loosening of the cage. In our series, most patients with class II and class III lesions were reconstructed with the Burch-Schneider cage, multiple screws fixed to the remaining intact ilium, cement augmentation and total hip arthroplasty. There was no implant loosening or implant revision in our series. These results lead to our belief that if stable fixation was achieved by using the protusio cage before cementation, adding Steinmann pins might not be necessary. Without using Steinmann pins in class III lesions, stable construct would be achieved by fixing multiple screws toward the sacroiliac joint where the bone stock is usually preserved. Sometimes the hip center might be slightly elevated because of compression forces from screws used to achieve stable fixation; however, we found this was not a significant problem regarding patient ambulation. However, when a stable construct was not achieved by the described method, adding Steinmann pins or screws is still highly recommended to enhance stability. Radiographs of the patient in Figure 2 demonstrate the addition of some long screws to enhance the stability; we aimed one screw toward the sacroiliac joint and the other screws to the lesion. Retrospectively reviewed, we believed that adding these screws might add little strength to the construct. The reason that the construct did not fail should be that it was sufficiently stable before adding those screws. Our result was similar to the findings from Kunisada [7] which showed no implant loosening from 25 patients with class III lesions and Steinmann pins were inserted in only 7 patients.

Reconstruction with hemipelvic endoprosthesis would be a good alternative with stronger construct and acceptable functional outcome [8, 9]. However, high complication rates, in up to 75% of patients, have been reported and these mostly involved wound-related problems [8, 9]. With the higher cost of these endoprostheses, higher complication rates and relatively limited life expectancy of these patients, careful consideration must be taken when choosing hemipelvic endoprostheses.

Our results showed that almost all patients benefit from surgical reconstruction. Sixteen bed-bound and three wheelchair-bound patients and one household ambulatory patient became seven community and twelve household ambulators. One patient remained bed-bound because of pain at other metastatic sites. The average ECOG performance status scores improved from 3.1 preoperatively to 1.7 postoperatively (*P* < 0.001). Pain scale was also improved from 8.4 before the operation to 2.2 after the operation (*P* < 0.001). Besides, patient performance was dramatically improved, especially in self-care activity, which is the most important activity formostpatients.

Our dislocation rate (5%) was comparable to other studies [6, 7, 10]. We tried to reduce this complication by tightly repairing the hip capsule as well as using a larger femoral head and preserving the continuity of the abductor mechanism. In two reconstruction cases where the hip capsule was not clearly identified and was removed with the tumor, a vascular graft was used to replace the original capsule and was tightly sutured with the remaining capsule until the graft and the remaining capsule circumferentially covered the hip joint as described by Bickels *et al*. [11]. One patient developed superficial infection (5%) at 2 weeks after the surgery and was treated successfully with wound irrigation. There was no deep infection. The lower rate of infection compared to some studies [4, 5] could pertain to our reconstructions, which were mostly performed before radiation and chemotherapy treatment.

The mean survival time in our patient population was 11 months, which is within the range reported by other series [1, 5, 12]. The difference in patient survival would be due to diversity of the patient population. Half of our patients in this study included lung carcinoma and hepatocellular carcinoma patients where the prognosis is not as good as other cancers, such as breast cancer, which were more common in other series [13].

The limitations to this study include the small numbers in the patient populations, retrospective data collection, diversities in patient comorbidities and their individual treatment that would affected their postoperative ambulatory status.

## Conclusion

In spite of the limited life expectancy of patients with periacetabular metastasis, reconstruction using anti-protusio cages, screws and cemented hip replacement is significantly useful for improving their quality of life; creating independent ambulation and controlling pain. With careful, thoroughly preoperative preparation and proper surgical reconstruction, these patients in our study were managed successfully with fewer complications.

## Consent

Written informed consent was obtained from the patient for the publication of this report and any accompanying images.
